# Phytochemical Characterization and Antimicrobial Properties of a Hydroalcoholic Extract of *Tristerix corymbosus* (L) Kuijt, a Chilean Mistletoe Species Hosted on *Salix babylonica* (L)

**DOI:** 10.3390/antibiotics15010105

**Published:** 2026-01-21

**Authors:** Alejandro A. Hidalgo, Sergio A. Bucarey, Beatriz Sepúlveda, Sebastián Cumsille-Escandar, Alejandro Charmell, Nicolás A. Villagra, Andrés Barriga, Consuelo F. Martínez-Contreras, Jorge Escobar, José L. Martínez, Maité Rodríguez-Díaz

**Affiliations:** 1 Escuela de Química y Farmacia, Facultad de Medicina, Universidad Andres Bello, Santiago 8370134, Chile; alejandro.hidalgo@unab.cl (A.A.H.); sebastian.cumsille@unab.cl (S.C.-E.); a.charmelljameson@uandresbello.edu (A.C.); c.martinezcontreras@uandresbello.edu (C.F.M.-C.); 2 Departamento de Ciencias Biológicas Animales, Facultad de Ciencias Veterinarias y Pecuarias, Universidad de Chile, Santiago 8820808, Chile; sbucarey@uchile.cl; 3 Departamento de Ciencias Químicas, Facultad de Ciencias Exactas, Universidad Andres Bello, Santiago 8370134, Chile; bsepulveda@uc.cl; 4 Departamento de Ciencias Biológicas, Facultad de Ciencias de la Vida, Universidad Andres Bello, Santiago 8370134, Chile; nicolas.villagra@unab.cl; 5 Centro de Estudios Para el Desarrollo de la Química (CEPEDEQ), Facultad de Ciencias Químicas y, Farmacéuticas, Universidad de Chile, Santiago 8380492, Chile; anbarr@ciq.uchile.cl; 6 Laboratorio de Química Biológica, Facultad de Ciencias, Pontificia Universidad Católica de Valparaíso, Valparaíso 2340000, Chile; 7 Departamento de Ingeniería Metalúrgica, Facultad de Ingeniería, Universidad de Santiago de Chile, Estación Central, Santiago 9160000, Chile; 8 Departamento de Química, Escuela de Química, Facultad de Ciencias Naturales, Matemática y del Medio Ambiente, Universidad Tecnológica Metropolitana, Santiago 7800003, Chile

**Keywords:** *Tristerix corymbosus*, mistletoe, antimicrobial plant extract, antimicrobial lipidic compounds

## Abstract

**Background/Objectives:** The genus *Tristerix* comprises at least ten species, found from southern Chile to Colombia in South America. In Chile, several species of these hemiparasitic plants are known as quitral or quintral. Quitral, mainly *T. corymbosus* (syn. *T. tetrandus*), is used in alternative medicine for its anti-inflammatory, digestive, hemostatic, hypocholesterolemic, and wound-healing properties. This study investigates the phytochemical composition and antimicrobial properties of *T. corymbosus*. **Methods:** A hydroalcoholic extract of *T. corymbosus* was prepared from leaves and small branches. The addition of methanol, on the extract, produced precipitation allowing us to isolate a methanol-soluble fraction, a brown powder obtained after filtration, and a tar-like residue remaining in the flask. These fractions were resuspended and tested for antimicrobial activity. **Results:** All fractions showed activity against *Streptococcus pyogenes*, but not *E. coli*. The brown powder exhibits the strongest potency against Gram-positive bacteria, some Gram-negative and *C. albicans*. HPLC-MS analysis revealed presence of lipidic compounds with surfactant properties. **Conclusions:** The abundant lipidic molecules present in the analyzed fraction likely account for the antimicrobial effects through affecting membrane structure of microorganisms supporting the traditional wound-healing uses of *T. corymbosus* in ancestral medicine.

## 1. Introduction

An enigmatic group of plants, the so-called mistletoes have been described in the literature. Mistletoes are a type of parasitic plant comprising around 1600 species grouped in four families, *Loranthaceae*, *Misodendraceae*, *Santalaceae*, and *Viscaceae*, among which the *Loranthaceae* family is the largest, with 900 or more species. These plants are vegetables that, unable to satisfy their nutritional demands on their own, obtain the nutrients they require at the expense of another plant, called a host [1,2].

**Figure 1:**
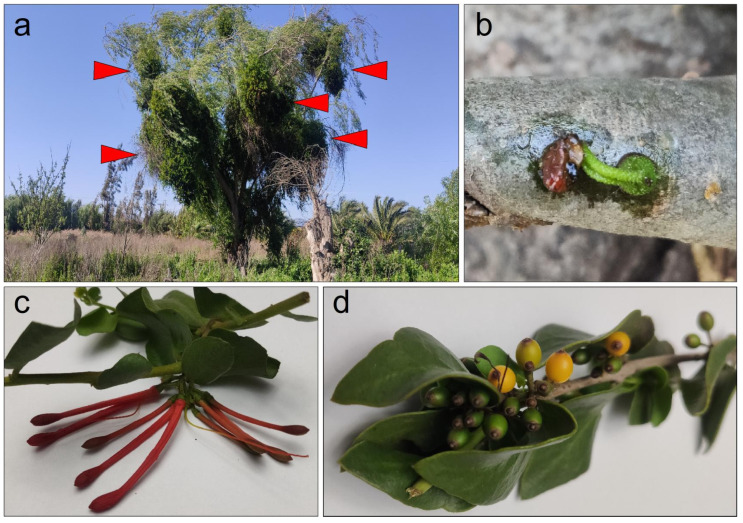
*T. corymbosus* on *Salix babylonica*. Locally known as quintral or quitral *T. corymbosus* establish a pernicious parasitic relationship with introduced species such as willow and poplar but long-term, less-invasive parasitic relationship with several native species. (**a**) *T. corymbosus* hosted in *S. babylonica* forms massive growth that in the long term kills the three. Red arrowheads indicate sites of *T. corymbosus* growth on its host. (**b**) During germination, *T. corymbosus* seeds stick to branches and start producing a haustorium that penetrates tree’s tissues. (**c**) The Chilean mistletoe *T. corymbosus* produces red inflorescences organized in corymbs, from January through September. (**d**) Detail of ripe and raw fruits of *T. corymbosus*.

The genus *Tristerix* comprises up to 13 species described as hemiparasitic plants belonging to the Loranthaceae family that grow only in South America from Chile and Argentina in the south to Colombia and Ecuador by the north of South America [2]. Two of these species grow in Chile. The *Tristerix aphyllus*, which grows on cacti, while the *Tristerix corymbosus* species grows on a wide variety of hosts, are among the most common, including poplar, huayún, willow, and olive trees (Figure 1a).

*T. corymbosus* (sin. *T. tetrandus*), the most common species of Chilean mistletoe, is typically found between latitudes 30° S and 40° S [1]. It is characterized by its striking red or orange tubular hermaphroditic flowers, which attract honeybees and hummingbirds for pollination. Its fleshy fruits are consumed and dispersed by birds, with seeds adhering to tree branches through mucilaginous sap after digestion (Figure 1c,d).

Germination begins when a haustorium develops, penetrating the tree’s endophytic system. *T. corymbosus* often parasitizes poplar and willow trees in central and southern Chile, which are more susceptible than native species, and can lead to the collapse of infested trees over time (Figure 1a,b).

Quitral (also known as quintral, cutral, ñipe, or liga) is one of the species used by the Mapuche people for its healing properties [3]. In traditional medicine, it has long been utilized for its antioxidant, astringent, hemostatic, hypocholesterolemic, wound-healing, digestive, and anti-inflammatory activities [3]. Unlike its European relative, *Viscum album* (mistletoe) [4,5,6,7], *T. corymbosus* has not been extensively studied for use in phytotherapeutics within complementary and alternative medicine (CAM). However, a limited number of studies have described the phytochemical composition and biological activities of *T. corymbosus* and related native species.

Simirgiotis and colleagues first described the phenolic compounds of Chilean mistletoe (*T. corymbosus*), endemic to Chile’s VIII Region and parasitic on poplar trees. This study identified 36 compounds, 30 of which were detected in mistletoe leaves, including caffeic acid derivatives, procyanidins, and various flavonoids. In addition, six anthocyanins were detected in the flowers. Significant differences were observed between leaves and flowers in both total phenolic content and antioxidant activity [8].

Recently, Torres et al. (2019) [1] investigated the antioxidant properties of *T. corymbosus* grown on three different host plants. This study revealed the phytochemical composition of various extracts and plant parts, such as glycosides, sterols, terpenoids, and quinones, in addition to high levels of total phenols and total flavonoids. Additionally, they reported their reducing power [1]. While Fernandez Moreno et al. (2025) described methanolic and chloroform extracts of *T. corymbosus* to be rich in flavonoids, possessing antioxidant activity, active against Gram-positive bacteria and with reasonable toxicity [9].

In this work, we studied the phytochemical composition and antimicrobial activity of a hydroalcoholic extract of *T. corymbosus* collected in central Chile.

## 2. Results

### 2.1. Different Types of Secondary Metabolites Are Present in the Hydroalcoholic Extract of Leaves, Flowers, and Fruits of T. corymbosus

The plant powder was soaked in 70% ethanol, as described in the Section 4. In a preliminary effort to find antimicrobial activity, small-scale (50 or 100 mL) hydroalcoholic extracts were prepared from the leaves, flowers, and fruits of *T. corymbosus*. Before evaluating its biological activity, a qualitative analysis of the phytochemical composition of *T. corymbosus* was conducted. The results suggested the presence of six compound families in the leaf extract, four in the flower extract, and eight in the fruit extract, exhibiting the greatest chemical diversity. All extracts tested positive for steroids and terpenes in the Liebermann–Burchard test, as well as for tannins and phenolic compounds in the ferric chloride test. As expected, carbohydrates were detected only in fruit extracts. Detailed qualitative composition data are presented in Table 1.

### 2.2. Preparation of Hydrosoluble Fraction from a Hydroalcoholic Extract from T. corymbosus

Plant powder was soaked in 70% ethanol (in water *v*/*v*) as outlined in the Section 4. In a preliminary effort to investigate antimicrobial properties, small-scale (50 or 100 mL) hydroalcoholic extracts were generated from the leaves, flowers, and fruits of *T. corymbosus.*

The extracts were rotovaped and tested for antimicrobial effects against *E. coli* and *Streptococcus pyogenes* (see Table 2) using an agar diffusion assay as described in detail below in Section 2.3 and Section 4.4. In this case, only 2 bacteria were utilized, a Gram-negative and a Gram-positive to screen for the fraction with the higher activity.

**Figure 2:**
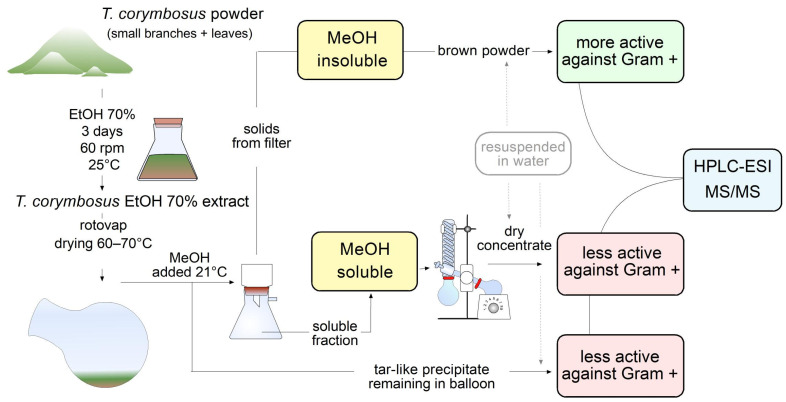
Workflow leading to evaluation of antimicrobial activity and phytochemical characterization of hydroalcoholic extract of *T. corymbosus*. Extracts were prepared in 70% ethanol and rotovaped at 60–70 °C. To keep drying the extract, methanol was added, producing separation of the extract in a soluble fraction, a brown powder and a tar-like residue. The brown powder turned out to be more active and was further characterized by its antimicrobial activity and phytochemically using HPLC-MS. The three fractions were resuspended in sterile water for antimicrobial activity testing.

Because antimicrobial effect was higher in leaves extract against Gram-positive *S. pyogenes* and because leaves are abundant and easy to collect, it was decided to prepare a higher scale hydroalcoholic extract starting with 100 g of leaves powder in 1 L of 70% ethanol. The extract was vac-filtered and rotovaped. Once the extract was reduced to around 100 mL of mostly water, 300 mL of methanol was added to keep rotovaping at 70 °C to extract remaining water. Incidentally, the addition of methanol produced a muddy precipitate which was filtered, dried at 37 °C and kept as a brown powder. At the same time, a tar-like insoluble fraction remained at the bottom of the original flask; such an insoluble fraction was also kept for further characterization. The flowthrough fraction (in methanol and water) was rotovaped to dry. The three fractions were soluble in 70% ethanol or water and were kept in an amber-color flask for further characterization. Schematic representation of steps followed from extraction to antimicrobial activity essays and identification of molecules is shown in Figure 2.

### 2.3. Hydrosoluble-Powder Fraction Obtained from the Hydroalcoholic Extract of T. corymbosus Shows Potent Antimicrobial Effects

**Figure 3:**
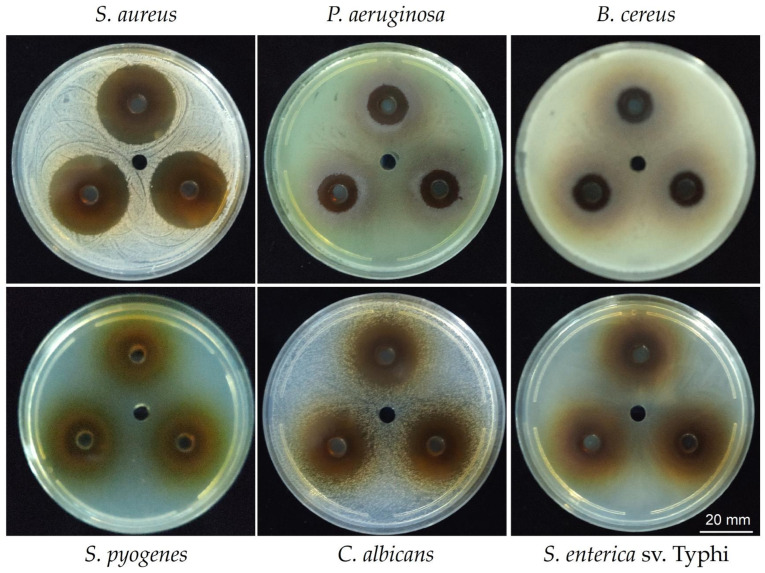
Inhibition haloes produced by the HSF of *T. corymbosus*. A well diffusion assay was used to evaluate antimicrobial effects of the HSF of *T. corymbosus* on Gram-positives, Gram-negatives, and *Candida albicans*, a yeast. The figure shows some of the tested bacteria in technical triplicates and the vehicle in the central well.

To test the antimicrobial activity of the hydrosoluble fraction (HSF) obtained from the ethanolic extract of *T. corymbosus* the well diffusion was performed by adding 35 µL of HSF (150 mg/mL) in each of a 6 mm well, formed in a Petri plate. As shown in Figure 3, the HSF presents inhibitory effects against Gram-positives, Gram-negatives, and yeast. The results are summarized in Table 3 where the higher effect is evidenced for the Gram-positive *S. aureus* and the yeast *C. albicans*. Importantly, the extract also presented activity against *Pseudomonas aeruginosa* and *Salmonella enterica* serovar Typhi.

Antimicrobial activity of HSF was also quantitatively evaluated by evaluating minimal inhibitory concentration. Such procedure confirmed a potent effect against *S. aureus* and *C. albicans*, as well as antimicrobial effects observed in the diffusion assay for the other microorganisms. In the case of Gram-negatives, the antimicrobial effects depend on the species and even the serovar, as the HSF is somehow active against *Salmonella enterica* serovar Typhi but less active against *S. enterica* serovar Typhimurium.

### 2.4. Antimicrobial Effects of the Hydrosoluble-Powder Fraction of T. corymbosus in Clinical Isolated Bacteria

**Figure 4:**
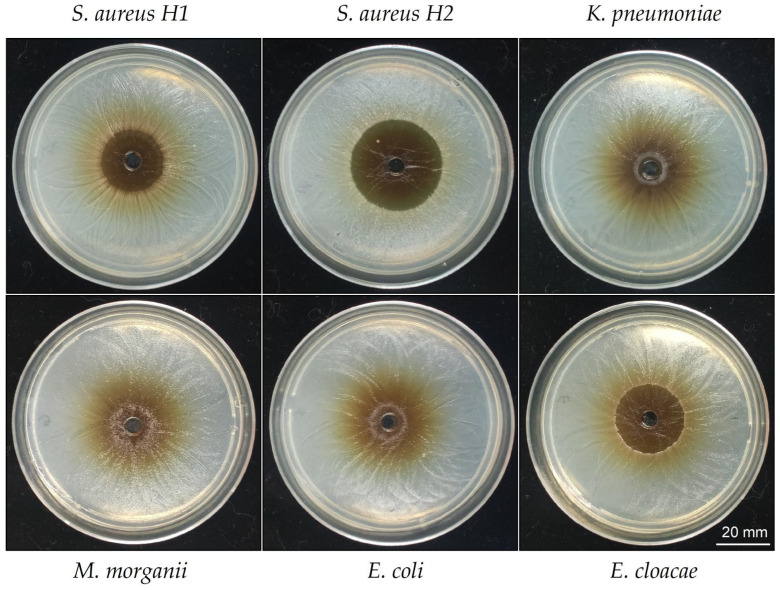
Inhibition haloes produced by the HSF of *T. corymbosus* in clinical isolated bacteria. A well diffusion assay was used to evaluate antimicrobial effects of the hydro-soluble extract of *T. corymbosus* on clinical isolated *S. aureus* and clinical isolated Gram-negatives such as *K. pneumoniae*, *M. morganii*, *E. coli*, *E. cloacae*, and *E. aerogenes*. The antibiogram for all clinical isolated tested and their antibiotics resistant profile is provided in Table 4. The experiments were performed at least three times.

To test the ability of the HSF to inhibit growth of clinical isolated bacteria, antimicrobial activity was tested against *S. aureus* and four Gram-negative clinical isolated bacteria, ranging from resistant to multiresistant, see detail in Table 4. The *S. aureus* strains presented different degrees of sensitivity to HSF. When it comes to Gram-negative bacteria, only *E. cloacae* showed a potent inhibition. Importantly, the HSF presents antimicrobial activity against resistant and multiresistant bacteria that spread between high to moderate (see Figure 4 and Table 4).

### 2.5. LC-MS Analysis and Identification of T. corymbosus Ethanolic Extracts

**Figure 5:**
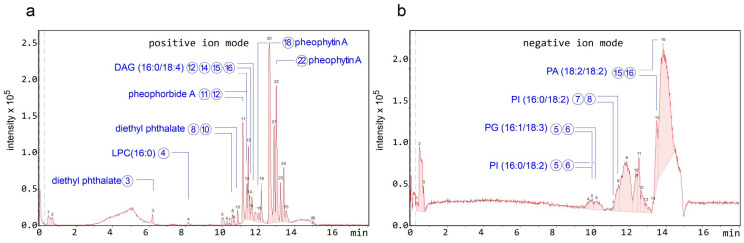
Molecular profile of the HSF obtained from the ethanolic extract of *T. corymbosus*. Chromatogram obtained from the negative polarity detection mode (**a**) and Chromatogram obtained for positive polarity detection mode (**b**) of the HSF from *T. corymbosus*. The numbered peaks were tentatively identified using a spectrometric database.

Figure 5a shows the chromatogram obtained in positive polarity, in which a high density of peaks was observed, with greater intensity in the most hydrophobic region of the gradient. In negative polarity, a similar trend was observed, although the peaks exhibited low chromatographic resolution (Figure 5b). The tentative identification of some of the peaks is detailed in Table 5, which also includes the molecular formula, theoretical *m*/*z* (*m*/*z*-theo) and experimental *m*/*z* (*m*/*z*-exp) signals, the mass error in ppm between *m*/*z*-theo and *m*/*z*-exp, the modified cosine (MQ) score provided by GNPS, and the chemical classification of the identified compounds.

In positive polarity, the compounds identified corresponded mainly to lipids such as Lysophosphatidylcholine LPC (16:0) and diacylglycerol DAG (16:0/18:4), as well as tetrapyrrolic pigments characteristic of chlorophyll degradation, including 3,10S-hydroxypheophorbide A, pheophorbide A, pyropheophorbide A, and pheophytin A. 13-Docosenamide, a fatty acid amide, was also identified, along with diethyl phthalate, a phthalic acid ester that is likely an exogenous contaminant.

**Figure 6:**
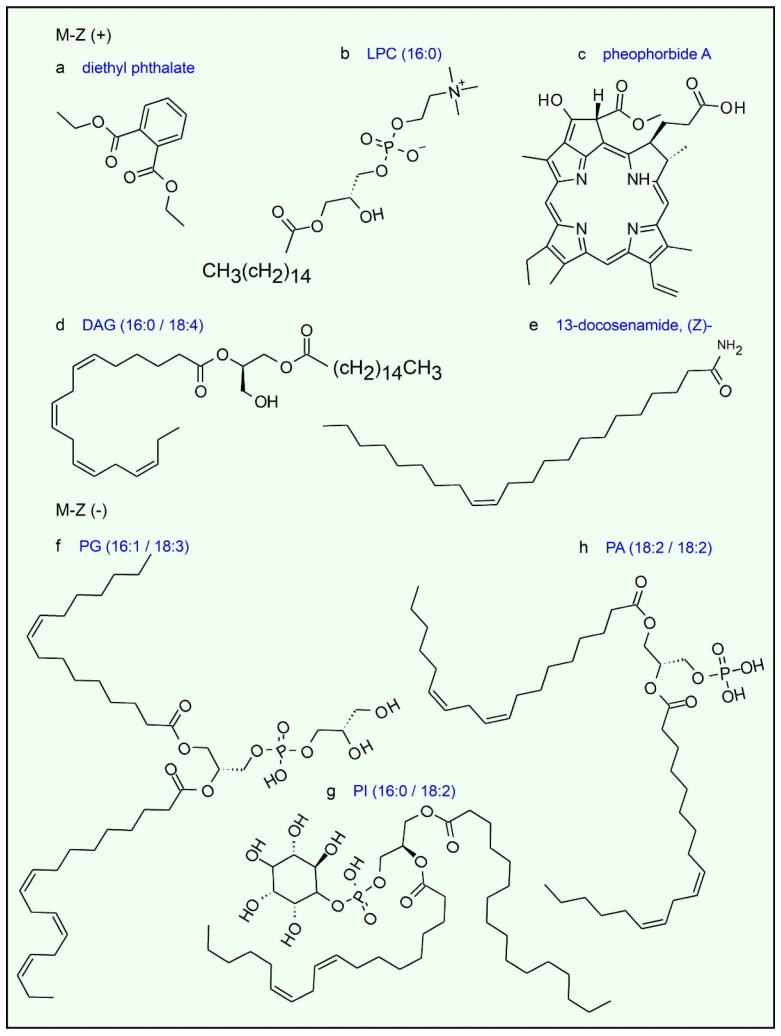
HSF obtained from *T. corymbosus* is rich in lipidic compounds. Identified molecules of positive and negative ion modes include (**a**) diethyl phthalate a compound regarded as both synthetic and naturally occurring. (**b**) Lysophosphatidylcholine (LPC) is a acylphosphocholine. (**c**) Is a porphyrinic compound representative of several related molecules detected in the extract. (**d**) shows the most representative diacylglycerol molecule corresponding to DAG (16:0/18:4) formula. (**e**) (*Z*)-13-docosenamide known as erucamide, a natural molecule derived from erucic acid found in plants. For (**d**,**f**–**h**), the formulae with the most representative in saturations are shown, albeit we cannot specify the precise position of the double bonds in the acyl-moiety of each chemical species.

In negative polarity, the identifications corresponded mainly to glycerophospholipids, including phosphatidylglycerols such as PG (16:1/18:3), phosphatidylinositols such as PI (16:0/18:2), and phosphatidic acids such as PA (18:2/18:2), all of which are typical components of biological membranes. The predominance of these phospholipids in negative mode is consistent with their anionic nature, derived from the presence of phosphate groups that ionize efficiently under these conditions. Co-elution of compounds was observed in both polarities, and several peaks shared the same identification, likely due to the presence of isomers. Formulae of most representative compounds found in the HSF are depicted in Figure 6.

### 2.6. The Hydro-Soluble Extract of T. corymbosus Produces Bacterial Dead Mainly by Affecting Membrane Integrity

**Figure 7:**
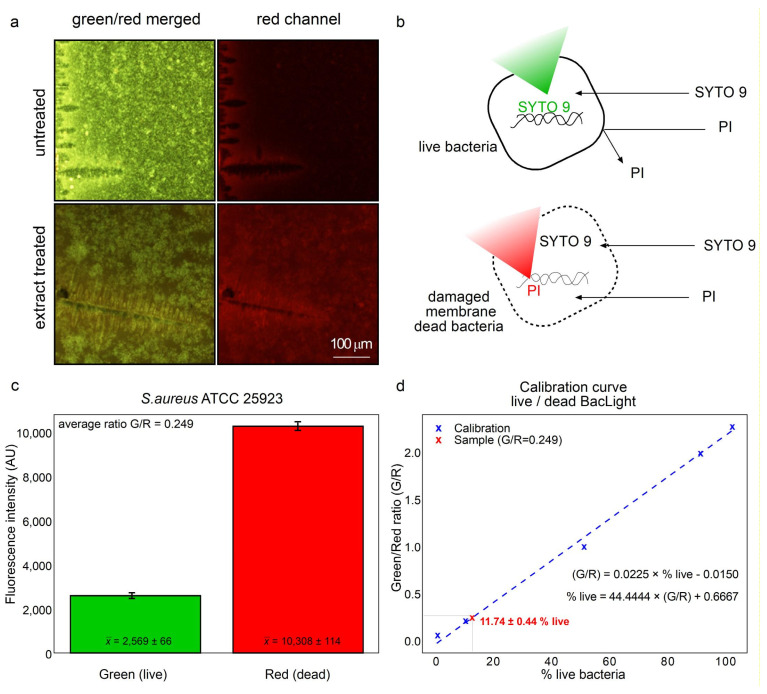
HSF obtained from *T. corymbosus* induces bacterial death by affecting membrane stability. Incubation with 7.5 mg/mL of HSF was sufficient to inhibit growth of *S. aureus* by affecting membrane integrity. Treatment with HSF promotes permeability and kills cells facilitating entry of propidium iodine (PI) resulting in increased red fluorescence (**a**). As PI enters the bacterial cell, it displaces SYTO 9, decreasing green fluorescence (**b**). The capabilities of PI and SYTO 9 of permeating dying and intact cells allow calibrating the ratio of green/red fluorescence versus percentage of live cells, to finally estimate viable cells in around 11.7% ± SD and the remaining 88.3% with membrane damage (**c**,**d**). The experiment was repeated three times.

Because of the high content of lipidic chemical species in the hydro-soluble extract it was hypothesized that membrane damage could be important for the antibacterial effects. Therefore, the LIVE/DEAD BacLight Bacterial Viability Kit was used (Figure 7). The protocol includes the use of two dyes that stain nucleic acids. The SYTO 9 dye penetrates all cells staining green cells with intact membranes. While propidium iodine only penetrates cells with damaged membranes ousting SYTO 9, resulting in red fluorescence, as depicted in Figure 7a,b. The green/red fluorescence ratio was used to determine the percentage of viability and membrane integrity. As seen in Figure 6, in untreated bacteria, green fluorescence predominates (compare Figure 7a upper with lower panel). Analogously, in extract-treated bacteria red fluorescence predominates compared to the untreated group (compare Figure 7a lower with upper panel). Figure 6b depicts the affinity of SYTO 9 staining toward nucleic acids, as it penetrates inside intact cells. While SYTO 9 also penetrates through the membrane of damaged cells, SYTO 9 is displaced from nucleic acids by PI staining which possesses higher affinity and emits red light. Higher red fluorescence compared to green (Figure 7c) is indicative of 11.9% live cells as the viability is extrapolated from a calibration curve (Figure 7d). Therefore, over 88% of cells were damaged at the cellular membrane.

## 3. Discussion

There are few reports describing the phytochemical composition of *T. corymbosus* extracts and related species. This work describes a fractionated extract with important antimicrobial effects mainly against Gram-positive bacteria such as *S. aureus*, *S. pyogenes*, and *B. cereus*, but also antimicrobial effects on some enterobacterales such as *S.* Typhi. In addition, antimycotic activity was observed for *C. albicans*. Importantly, the antimicrobial effects were confirmed for all clinical isolated *S. aureus* tested and observed for enterobacterales such as *E. cloacae* and with less intensity for *Klebsiella* and *Morganella*. The level of sensitivity to the HSF was especially higher for *S. aureus* for both, the standard, or clinical isolated. The fact that plant extracts are more active against Gram-positive find support in the literature regarding the magnitude of the effects against Gram-positive bacteria, number of plants with effects on Gram-positive versus Gram-negative, and the spectrum on those bacteria [10,11,12]. In most cases the antimicrobial effect of plant extracts on Gram-positive bacteria are attributed to compounds such as polyphenols, flavonoids and terpenes that easily cross the plasmatic membrane of Gram-positive bacteria whose lack an outer membrane and the lipopolysaccharide. However, the HS fraction of the hydroalcoholic extract of *T. corymbosus* described here is rich in lipidic compounds such as acyl-glycerols, acyl-phosphoglycerol, acylphosphocholine and acylamides. In addition, porphyrinic compound derived from chlorophyll and diethyl phthalate were found.

Lysophosphatidylcholine (LPC) which is abundant in mammalian cells, serves as a mediator of inflammation and trigger of apoptosis has also been described as a cell signal in plants. The related lysophosphatidylcholine 17:1 was found on the leaf surface of wild potato *Solanum bulbocastanum* and, as confirmed by in vitro assays, would be responsible for inhibiting the fungi *Phytophthora infestans* [13]. The antimicrobial effects of lysophosphatidylcholine are widely described against bacteria including direct effects against methicillin resistant *S. aureus* or as a coadjutant increasing efficacy of colistin in Gram-negatives [14,15,16]. Erucamide has been described by its properties reducing growth but also virulence of the plant pathogen *Ralstonia pseudosolanacearum* [17]. In a different study, erucamide was present in extracts of *Bacillus megaterium* with antibacterial effects against plant pathogens *Agrobacterium tumefaciens*, *Erwinia carotovora*, and *Ralstonia solanacearum* [18]. While fatty acids and monoglycerides has been study for their potential inhibit viral and bacterial infections [19,20,21,22,23,24,25]; studies in PG (diacylphosphogicerides), PI (phosphatidylinositol), and PA (diacyl phosphatidic acids) are scarce and refer to some in vivo and in vitro effects inhibiting infection of some viruses [26,27,28].

**Figure 8:**
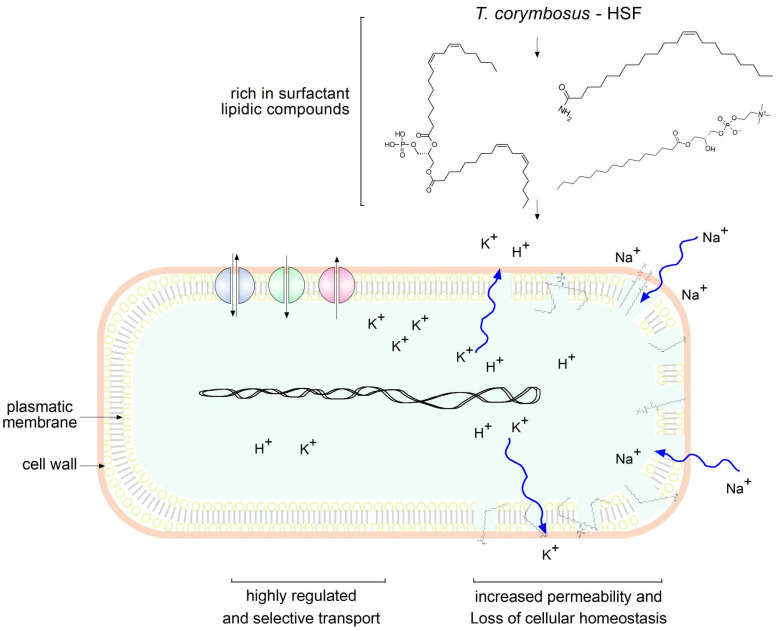
Lipidic compounds found in HSF obtained from *T. corymbosus* alter membrane permeability. According to results and the literature, lipidic compounds found in plant extracts exert antimicrobial effects by increasing permeability. Zig-zag curved arrows indicate free flow of ions as damage affects cellular homeostasis.

The predominant presence of lipidic compounds in the HSF leads to hypothesizing that the antimicrobial effects may be due to the disruption of membrane integrity or, at least, changes in permeability, as depicted in Figure 8. Therefore, studies to determine permeability, because of membrane damage, were performed and confirmed the hypothesis in *S. aureus* which resulted very sensitive in the antimicrobial assays (Figure 7). This is a strong observation, because HSF presented significant antimicrobial effects not only on a standard strain, but also in clinical multi-resistant isolates of *S. aureus* (Figure 4 and Table 4).

The described HSF was rich in lipidic and porphyrinic compounds. However, the whole original extract was rich in flavonoids, anthraquinones, tannins, and phenolic compounds between other species. Therefore, results from a previous study are consistent with this composition [8]. The precipitation induced by adding methanol allowed purifying a fraction rich in lipidic compounds with surfactant activity, since the precipitate is hydro soluble. Such features are consistent with the composition of polar lipidic species, as evidenced by mass spectroscopy studies, Table 5 and Figure 5.

The antimicrobial profile observed for the hydrosoluble fraction (HSF) of *T. corymbosus* may reflect a synergistic contribution among the lipidic and minor phenolic constituents identified by LC-MS. It is plausible that the amphiphilic molecules such as phosphatidylinositol (PI), phosphatidylglycerol (PG), and lysophosphatidylcholine (LPC) act not only through direct membrane perturbation but also by modulating bacterial stress responses and permeability, facilitating the entry of other bioactive molecules. Similar synergistic effects between lipidic surfactant-like compounds and polyphenols have been reported in other plant-derived extracts, enhancing bactericidal activity particularly against Gram-positive pathogens [10,29]. Furthermore, the moderate effects against selected Gram-negative strains such as *S.* Typhi and *P. aeruginosa* suggest that lipid-based molecules could partially overcome the outer membrane barrier, possibly by increasing lipopolysaccharide fluidity or interacting with membrane proteins involved in antibiotic resistance [14,16].

The ability of HSF to inhibit multidrug-resistant *S. aureus* isolates is particularly relevant in the context of rising antibiotic resistance. Plant-derived lipidic molecules, such as lysophospholipids and long-chain amides (e.g., erucamide), reduce bacterial virulence and biofilm formation and to sensitize resistant strains to conventional antibiotics [17,18]. Accordingly, the lipid-rich composition of *T. corymbosus* extract may confer a dual mechanism of action, involving direct membrane disruption and adjuvant effects that enhance antibiotic susceptibility. These findings support the potential development of combinatorial strategies using *T. corymbosus* fractions as natural adjuvants, warranting further pharmacodynamic and mechanistic studies.

The antimicrobial effects might explain in part some of the uses; however, we need to consider first the diversity of *T. corymbosus* regarding their host and the subtractions of metabolites *T. corymbosus* make as it fuses its vascular system to the vascular system of the host. Such differences have been addressed to establish differences between *T. corymbosus* hosted by *Aristotelia chilensis* and poplar [1]. In addition, *T. corymbosus* is one of at least three species commonly known under the name of quitral, and possibly all may have been used in traditional medicine.

## 4. Materials and Methods

### 4.1. Plant Material

**Figure 9:**
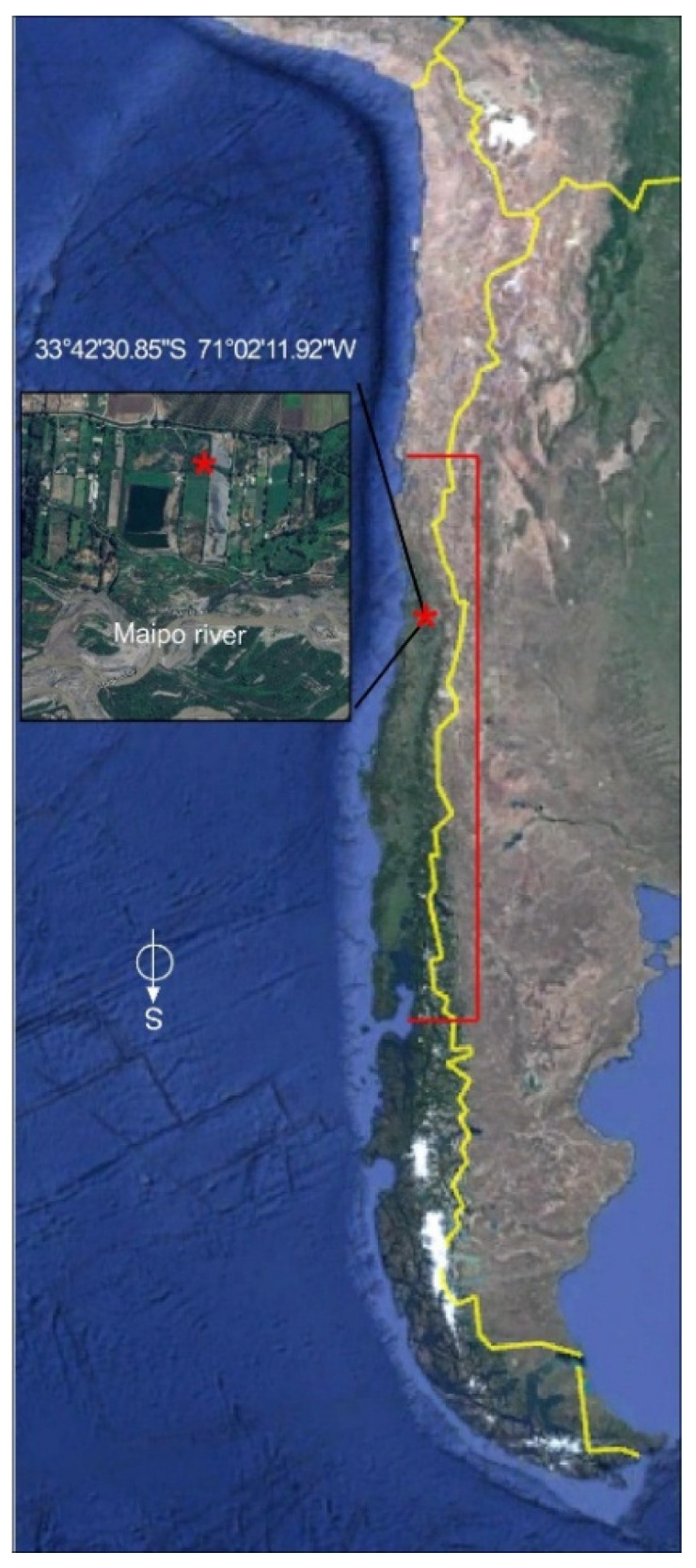
Place of collection of *T. corymbosus* on *S. babylonica*. Locally known as quintral or quitral, *T. corymbosus* establishes a pernicious parasitic relationship with introduced species such as willow and poplar but long term less invasive parasitic relation with several native species. Samples were collected in El Paico Town, El Monte, RM, Chile. The red bracket indicates the distribution of *T. corymbosus* in Chile. Red asterix indicates the precise location where samples were taken. Yellow lines are the country’s limits.

Leaves and small branches of *T. corymbosus* were collected in El Paico, El Monte, RM, Chile (33°42′30.85″ S 71°02′11.92″ W), as shown in Figure 9. *T. corymbosus* was hosted in a *S. babylonica*. The collected material was validated by the specialist Scarlett Norambuena and deposited at the Herbarium of Facultad de Ciencias Químicas y Farmacéuticas, Universidad de Chile, under code number SQF#22.906. The leaves, flowers, fruits, and small branches of *T. corymbosus* were dried for 15 days at 21 °C and crushed down in a porcelain mortar.

### 4.2. Plant Material Extract

The extracts were prepared by a discontinuous method using 70% ethanol (Merck, Darmstadt, Germany) as dissolvent, as previously described [30]. In brief, 100 g of dry plant powder (flowers, fruits, or leaves and small branches together) was macerated with 1 L of ethanol (70% in water *v*/*v*) for 3 days at 60 rpm and 25 °C. Following, the extract was filtered and rotovaped from 60 to 70 °C. When the extract was reduced to around 100 mL of mostly remaining water, 300 mL of methanol (Merck, Darmstadt, Germany) were added to keep extracting remaining water. The addition of methanol produced a muddy precipitate that was kept at room temperature (21 °C) for 30 min to complete precipitation. The precipitate was vacuum filtered, while a tar-like insoluble fraction remained at the bottom of the original flask. The flowthrough fraction (in methanol and water) was rotovaped to dry. Finally, the three fractions were resuspended in 80% of ice-cooled methanol (*v*/*v*) for further characterization (e.g., HPLC-MS) or in sterile water or LB medium for antimicrobial activity testing. Schematic representation of steps followed from extraction to antimicrobial activity essays and identification of molecules is shown in Figure 2.

### 4.3. Phytochemical Characterization

To qualitatively pinpoint the main families of phytochemicals in the hydroalcoholic extract of *T. corymbosus* obtained from leaves, flowers, and fruits, standard phytochemical tests were performed [31,32]. The tests performed include aluminum chloride for flavonoids, Keller–Killiani for cardiac glycosides, foam formation for saponins, Bornträger for anthraquinones, fluorescence for coumarins, Liebermann–Burchard for steroids and terpenes, and Dragendorff for alkaloids, flavonoids, and phenolic substances [29]. Detailed explanations on performing these phytochemical tests have been outlined in the literature [33,34,35,36]. Also, these phytochemical tests have been previously summarized in the Supplementary Material presented by Rodríguez-Díaz et al. 2024 [30].

### 4.4. Antimicrobial Activity Assays by an Agar Diffusion Test

Mueller–Hinton agar (Millipore, Darmstadt, Germany) was prepared by pouring 20 mL of hot medium into 90 mm disposable Petri dishes. After solidification and drying in a culture cabinet, the agar surface was inoculated with a microorganism suspension (McFarland 0.5 in 0.9% NaCl) using a cotton swab and a rotaplate to obtain a uniform lawn. Wells (6 mm) were then punched and filled with 35 μL of either the extract (150 mg/mL of water) or the vehicle (water), as required by each assay. Inhibition zone diameters were measured in three directions after 16 h incubation. Description of the method was previously published [30]. All experiments were performed independently at least three times.

### 4.5. Minimum Inhibitory Concentration (MIC)

Base-two serial dilutions were prepared in either Potato-Dextrose broth (for yeasts) (Millipore, Darmstadt, Germany) or LB-Luria (for bacteria) (Millipore, Darmstadt, Germany). A flat-bottom 96-well plate was loaded in its first row with 200 μL of culture media containing 15 mg/mL of HSF in LB medium containing bacteria or yeast (from an overnight inoculum adding same volume to reach McFarlan 0.5 in saline buffer); therefore, each column contained a different microorganism. The rest of the wells were filled with 100 µL of diluted bacteria or yeast, with the microorganism corresponding to each column, before carrying out the base-two dilutions. Following 16 h of incubation at 30 °C, cultures’ turbidity was assessed by visual observation, the lower concentration capable of inhibiting growth was reported as the MIC [37]. The procedure was repeated at least three times without variations.

### 4.6. LC-MS Analysis

#### 4.6.1. Sample Preparation and LC-MS Analysis

The lyophilized HSF extract was resuspended in 80% ice-cooled methanol in water (*v*/*v*), passed through a 45µM PVDF filter (Millipore, Darmstadt, Germany) before injection. Samples were analyzed using an LC-MS system consisting of an Elute UHPLC chromatograph (Bruker Daltonik GmbH, Bremen, Germany) coupled to a Compact ESI-TOF mass spectrometer (Bruker Daltonik GmbH, Bremen, Germany) equipped with an electrospray ionization (ESI) source. Instrument control and data acquisition were performed using HyStar 4.0 software (Bruker Daltonik GmbH, Bremen, Germany). Chromatographic separation was carried out on a Kinetex C18 column (100 × 2.1 mm; Phenomenex Inc., Torrance, CA, USA). A 3 μL aliquot of each resuspended extract was injected using the following gradient program: 0.0–0.5 min, 12.0% B; 0.5–11.0 min, 12.0–99.0% B; 11.0–14.0 min, 99.0% B; 14.0–14.2 min, 99.0–12.0% B; and 14.2–18.0 min, 12.0% B. Mobile phase A consisted of 0.1% formic acid (*v*/*v*) in water, and mobile phase B consisted of 0.1% formic acid (*v*/*v*) in acetonitrile. The flow rate was set to 0.4 mL/min and the column temperature was maintained at 40 °C. Ionization was performed via electrospray at 4500 V, using nitrogen as nebulizing gas at 2.0 bar and 8 L/min, and as drying gas at 250 °C. Mass spectra were acquired in both positive and negative ion modes.

#### 4.6.2. Data Processing and Compound Identification

Chromatographic and spectral data were visualized and processed using DataAnalysis 4.4 (Bruker Daltonik GmbH, Germany). Prior to downstream analyses, the spectral data were calibrated using the calibration peak at tR 0.1 min within the *m*/*z* range 50–1300, corresponding to sodium/formate ion clusters. Calibrated mass spectral data were exported in mzXML format as centroid spectra for further analyses. Compound identification was performed using the Global Natural Products Social Molecular Networking (GNPS) platform (https://gnps.ucsd.edu) [38]. The mzXML files were submitted to the Library Search workflow using all available spectral libraries. The search parameters were a precursor ion mass tolerance of ±0.025 Da and a fragment ion mass tolerance of ±0.02 Da; default settings were used for all other parameters. Tentative identifications were accepted when the difference between the experimental *m*/*z* (*m*/*z*-exp) and the theoretical library *m*/*z* (*m*/*z*-theo) was ≤10 ppm and when the modified cosine (MQ) score supported an acceptable spectral match [38].

### 4.7. Analysis of Membrane Damage Using LIVE/DEAD BacLight Assay

Due to the abundance of lipidic molecules in the extract, with potential detergent activity, damage at the membrane level was evaluated using the LIVE/DEAD BacLight Bacterial Viability Kit (Molecular Probes Inc., Eugene, OR, USA) which combines two dyes that stain nucleic acids. SYTO 9 dye penetrates all cells, staining those with intact membranes predominantly green. Propidium iodide only penetrates bacteria with damaged membranes, displacing SYTO 9 and resulting in red fluorescence. Therefore, intact cells remain predominantly green, while membrane-damaged cells turn red. The green/red fluorescence ratio serves as a quantitative measure of bacterial membrane integrity and viability [39]. To quantify the damaged and intact bacterial cells, a culture of bacteria was exposed to 7.5 mg/L of the extract at 37 °C for 24 h. Fluorescence was measured using the Tube luminometer GloMax^®^ Multi JR detection system (Promega, Madison, WI, USA) using excitation wavelength at 480 and 490 nm and emission detected at 500 and 635 nm (green and red filters E6073 and E6074, respectively). The ratio of green/red fluorescence was used to interpolate the fraction of viable and damaged cells from a G/R versus percentage of viable cells produced by using killing bacteria with 70% isopropanol and then mixed with intact bacteria to produce a calibration curve with well-known proportions of killed/alive cells. Visualization of the assay was performed using a Nikon Eclipse E400 fluorescence microscope (Nikon Instruments, Tokyo, Japan) using excitation wavelengths 488. Visualizations were at 500 and 635 wavelengths.

## 5. Conclusions

In this study, a hydroalcoholic extract of *Tristerix corymbosus* was prepared, fractionated, and evaluated for its phytochemical composition and antimicrobial activity. The hydrosoluble fraction obtained after methanol-induced precipitation exhibited the strongest antimicrobial effects, particularly against Gram-positive bacteria, including standard and clinical isolates of *Staphylococcus aureus*, but also against some Gram-negative and *Candida albicans*.

Phytochemical screening and LC–MS analysis revealed that this active fraction was predominantly composed of lipidic compounds, including glycerolipids, glycerophospholipids, lysophosphatidylcholines, and fatty acid amides. Functional assays demonstrated that the antimicrobial activity of this fraction was associated with disruption of bacterial membrane integrity.

Overall, these results demonstrate that *T. corymbosus* contains bioactive lipid-rich fractions with significant antimicrobial activity, supporting its relevance as a source of natural antimicrobial compounds.

## Figures and Tables

**Table 1:** Qualitative determination of secondary metabolites in ethanolic extracts of *T. corymbosus*.

Compounds Tested	Assay	Leaves	Flowers	Fruits
alkaloids	Dragendorff	-	++	-
	Mayer	-	-	-
	Wagner	-	-	-
anthraquinones	Bornträger	+	-	+
Saponins	Foam formation	+	-	+
cardiac glycosides	Keller–Killiani	+	-	+
flavonoids	Shinoda	+	-	-
	Aluminum chloride	-	-	-
	Alkaline reagent test	+	-	+
reducing sugars	Fehling	-	+	+
steroids and terpenes	Liebermann–Burchard	+	+	+
	Salkowski	-	+	-
amino acids & proteins	Ninhydrin	-	-	-
tannins and phenolic	Ferric chloride	++	+++	+
carbohydrates	Molish	-	-	+
coumarins	Fluorescence under UV	-	-	-

One, two or three + signs indicate intensity of reaction compared across samples.

**Table 2:** Activity of the raw hydroalcoholic extract against *S. pyogenes* and *E. coli*.

Extract	Microorganism	Inhibition Haloes (mm)	Standard Error
leaves	*S. pyogenes*	28.9	3.1
	*E. coli*	6.0	0.0
flowers	*S. pyogenes*	13.7	2.5
	*E. coli*	6.0	0.0
fruits	*S. pyogenes*	20.3	1.7
	*E. coli*	6.0	0.0

Bacterial strains correspond to *S. pyogenes* ISP36900 and *E. coli* ATCC 25922.

**Table 3:** Antimicrobial activity of the hydroalcoholic extract of *T. corymbosus* against standard bacterial and yeast strains.

Microorganism	Inhibition Haloes (mm)	MIC (mg/L)
*Staphylococcus aureus*	29.7 ± 0.67	7.50
*Bacillus cereus*	16.0 ± 0.58	3.75
*Streptococcus pyogenes*	12.3 ± 0.33	7.50
*Escherichia coli*	9.0 ± 0.00	15.00
*S. Typhimurium*	9.3 ± 0.33	15.00
*S. Typhi*	19.0 ± 0.58	15.00
*Pseudomonas aeruginosa*	17.3 ± 0.33	15.00
*Candida albicans*	23.3 ± 0.33	7.50
*Cryptococcus. neoformans*	6.0 ± 0.00	15.00

The results are the average of three independent experiments ± standard deviation. MIC, minimal inhibitory concentration. *S. aureus* ATCC 25923, *B*. *cereus* ATCC 11778, *S. pyogenes* ISP36900, *E. coli* ATCC 25922, *Salmonella enterica* sv. Typhimurium 14028s (*S.* Typhimurium), *Salmonella enterica* sv. Typhi Ty2 (*S.* Typhi), *C. albicans* ATCC 90029, *C. neoformans* GM1. As a control, ampicillin was assessed against *S. aureus*. Five μg/well produced a 22 mm inhibition halo and the estimated MIC was 0.2 μg/mL. The experiments were performed three times with technical triplicates. Results are all statistically significant as *p* ≤ 0.01 after running the free ANOVA Calculator from Appinio web site. MIC values remained unchanged across replicates.

**Table 4:** Antimicrobial activity of the hydroalcoholic extract of *T. corymbosus* against a panel of clinical bacterial strains.

Microorganism	Inhibition Haloes (mm)	MIC (mg/L)	Resistance Found
*Staphylococcus. aureus* H1	29.33 ± 0.67	7.50	Amp^R^
*Staphylococcus aureus* H2	35.67 ± 0.58	7.50	Amp^R^, Cip^R^, Imi^R^
*Staphylococcus. aureus* H4	32.33 ± 0.58	7.50	Amp^R^, Cip^R^, Imi^R^
*Staphylococcus. aureus* H5	31.33 ± 0.58	7.50	Amp^R^, Cip^R^, Imi^R^
*Klebsiella. pneumoniae* H1	11.00 ± 0.00	15.00	Amp^R^
*Morganella morganii* H1	8.67 ± 0.88	15.00	Amp^R^
*Escherichia coli* H1	9.33 ± 0.58	15.00	Amp^R^, Cip^R^, Tet^R^
*Enterobacter cloacae* H1	26.67 ± 1.00	15.00	Amp^R^, Cip^R^, Tet^R^
*Enterobacter aerogenes* H6	6.00 ± 0.00	15.00	Amp^R^, Cip^R^, Imi^R^

The results are average of three independent experiments ± standard deviation. MIC, minimal inhibitory concentration. All strains correspond to clinical isolated bacteria maintained in the laboratory and collected by the Central Clinical Laboratory at Universidad the Chile during 2013–2016. Amp^R^, Cip^R^, Tet^R^, and Imi^R^ correspond to ampicillin, ciprofloxacin, tetracycline, and imipenem resistant phenotypes. Results are all statistically significant as *p* ≤ 0.01 after running the free ANOVA Calculator from Appinio web site. MIC values remained unchanged across replicates.

**Table 5:** HSF powder: identification of detected compounds.

**Positive polarity**									
**Peak**	**Rt (min)**	**Putative identification**	**Formula**	**Ion**	***m*/*z*-theo**	***m*/*z*-exp**	**Error (ppm)**	**MQ score**	**Classification**
3	6.2	Diethyl phthalate	C_12_H_14_O_4_	[M + H]+	223.0965	223.0959	2.6	0.966	-
4	8.2	LPC(16:0)	C_24_H_50_NO_7_P	[M + H]+	496.3398	496.3432	−6.9	0.949	Glycerophospholipids
8	10.6	3,10S-Hydroxypheophorbide A	C_35_H_36_N_4_O_6_	[M + H]+	609.2708	609.2654	8.8	0.746	Tetrapyrroles
10	10.9	3,10S-Hydroxypheophorbide A	C_35_H_36_N_4_O_6_	[M + H]+	609.2708	609.2710	−0.4	0.746	Tetrapyrroles
11	11.2	Pheophorbide A	C_35_H_36_N4O_5_	[M + H]+	593.2758	593.2750	1.4	0.963	Tetrapyrroles
12	11.4	DAG (16:0/18:4)	C_37_H_64_O_5_	[M + H]+	589.4827	589.4799	4.7	0.712	Glycerolipids
12	11.4	Pheophorbide A	C_35_H_36_N_4_O_5_	[M + H]+	593.2758	593.2764	−0.9	0.963	Tetrapyrroles
14	11.5	Pyropheophorbide A	C_33_H_34_N_4_O_3_	[M + H]+	535.2704	535.2704	−0.1	0.742	Tetrapyrroles
14	11.5	DAG (16:0/18:4)	C_37_H_64_O_5_	[M + H]+	589.4827	589.4861	−5.8	0.756	Glycerolipids
15	11.6	DAG (16:0/18:4)	C_37_H_64_O_5_	[M + H]+	589.4827	589.4807	3.3	0.758	Glycerolipids
16	11.7	DAG (16:0/18:4)	C_37_H_64_O_5_	[M + H]+	589.4827	589.4812	2.5	0.748	Glycerolipids
18	12.1	Pheophytin A	C_55_H_74_N_4_O_5_	[M + H]+	871.5732	871.5714	2.1	0.827	Tetrapyrroles
22	13.0	13-Docosenamide, (Z)-	C_22_H_43_NO	[M + H]+	338.3417	338.3413	1.3	0.830	Fatty acid amides
**Negative polarity**									
**Peak**	**Rt (min)**	**Putative identification**	**Formula**	**Ion**	*****m***/***z***-theo**	*****m***/***z***-exp**	**Error (ppm)**	**MQ score**	**Classification**
5	10.0	PG(16:1/18:3)	C_40_H_71_O_10_P	[M − H]−	741.4712	741.4652	8.1	0.715	Glycerophospholipids
5	10.0	PI(16:0/18:2)	C_43_H_79_O_13_P	[M − H]−	833.5186	833.5160	3.1	0.899	Fatty acids
6	10.5	PG(16:1/18:3)	C_40_H_71_O_10_P	[M − H]−	741.4712	741.4686	3.5	0.713	Glycerophospholipids
6	10.5	PI(16:0/18:2)	C_43_H_79_O_13_P	[M − H]−	833.5186	833.5140	5.5	0.928	Fatty acids
7	11.1	PI(16:0/18:2)	C_43_H_79_O_13_P	[M − H]−	833.5186	833.5163	2.7	0.884	Fatty acids
8	11.3	PI(16:0/18:2)	C_43_H_79_O_13_P	[M − H]−	833.5186	833.5163	2.7	0.896	Fatty acids
15	13.4	PA(18:2/18:2)	C_39_H_69_O_8_P	[M − H]−	695.4657	695.4592	9.4	0.842	Glycerophospholipids
16	13.6	PA(18:2/18:2)	C_39_H_69_O_8_P	[M − H]−	695.4657	695.4643	2.1	0.798	Glycerophospholipids

## Data Availability

The original contributions presented in this study are included in the article. Further inquiries can be directed to the corresponding author(s).

## Abbreviations

The following abbreviations are used in this manuscript:

HSF	Hydrosoluble fraction
LB	Lysogeny broth
PG	diacylphosphogicerides
PI	phosphatidylinositol
PA	diacyl phosphatidic acids
